# A Role for Public Health Training Through the Funding of University Departments of Rural Health

**DOI:** 10.1111/ajr.70191

**Published:** 2026-04-09

**Authors:** Sandra C. Thompson, Lisa Bourke

**Affiliations:** ^1^ Western Australian Centre for Rural Health, the University of Western Australia Geraldton Western Australia Australia; ^2^ Department of Rural Health University of Melbourne Shepparton Australia

## Abstract

**Aim:**

This commentary articulates the need for strengthened public health training and workforce development in rural and remote Australia, arguing that investment in preventive, community‐centred public health is essential to addressing longstanding health inequities. Rural and remote communities experience disproportionately high burdens of chronic disease, injury and premature mortality that are inadequately addressed within a health system that prioritises clinical care over population‐level approaches.

**Context:**

The authors situate this argument within the historical and contemporary context of rural disparities and rural health workforce policy. While University Departments of Rural Health (UDRHs) have long supported interprofessional rural training, the 2016 Rural Health Multidisciplinary Training (RHMT) programme narrowed eligible disciplines and excluded public health.

**Approach:**

The paper argues for strengthening rural public and preventive health within RHMT to build a locally embedded workforce capable of addressing the social, environmental and economic determinants of health. This includes supporting culturally safe, community‐led public health training in First Nations communities and recognising that local leadership and knowledge systems are central to effective solutions. The authors highlight systemic underinvestment in rural public health, noting limited health workforce, inadequate preventive infrastructure and a misalignment between national strategies and on‐the‐ground capacity.

**Conclusion:**

Achieving rural health equity requires renewed policy commitment to public health within the rural health training pipeline. Enabling public health students to train in rural areas alongside clinical students offers a cost‐neutral, immediately implementable step towards building a capable, community‐connected, prevention‐oriented rural health workforce.

## 1 Background

Australia's rural and remote communities are diverse, resilient and essential to Australia's economy and cultural identity, yet the 7 million residents living there (28% of Australians) experience higher rates of chronic disease, injury and premature death. Despite poorer health outcomes for rural and remote populations among both First Nations and non‐First Nations Australians, [1] the support for addressing poor health and underlying issues for these regions is inadequate. We argue that one of the most effective ways to address rural disparities is through targeted investment in preventive public health tailored to rural contexts. This demands investment in public health training and employment of an appropriately skilled workforce. We outline here the rationale for such investment to build a robust, locally embedded health workforce, and emphasise the role University Departments of Rural Health (UDRHs) can play to contribute to this critical rural functionality. This is an important issue for UDRHs, policymakers, the rural workforce and for health service improvement at a regional level.

The authors are established rurally based health academics who work for UDRHs funded with the key function of supporting student learning in rural areas. The UDRH programme started over 25 years ago and receives Australian government support for training the next generation of health professionals through opportunities for clinical learning, broader life exposures and to develop their skills in a rural setting. When first initiated, UDRHs included student training in public health as part of the broad group of skills for practice in rural settings [2]. Exposing students to the joys and challenges of rural health practice enables learning and experiences of the deficits in opportunities and services for some rural Australians. Such insights can transform career trajectories of emerging health professionals [3]. The authors call for public health to be included as a supported profession for UDRHs and other rural and remote training programmes.

## 2 Underinvestment in Rural Public Health

Documented disparities suggest substantial underinvestment in rural and remote public health, [4]. Rural areas are underfunded relative to their health burden, underinvestment which contributes to preventable hospitalisations, higher long‐term costs, and widening health inequities. The underspend in rural healthcare is calculated to be $6.55 billion, which equates to $848 per for each rural and remote Australian per year [5]. Higher rates of hospitalisation, injury and death, and poorer access to primary health care services for rural and remote residents [1] reflect the generally lower levels of preventive care and public health infrastructure. Service access does not match the population distribution; only a small fraction of the public health workforce is based in rural areas despite the higher burden of disease. Data on rural‐specific public health investment are limited [6], itself a barrier to equitable planning and funding. At present, the investment does not match the need which makes maintaining a strong, engaged cohort of rural health practitioners and researchers difficult, including for UDRHs [7].

## 3 The Value of a Public Health Approach

Public health is the practice of protecting and improving the health of people in communities through organised efforts. It focuses on disease prevention, health promotion, building health literacy and knowledge and ensuring access to healthcare services for all [8]. Public health is a discipline in its own right with both undergraduate and postgraduate training available. Rather than treating individuals, public health professionals take a population‐level approach, addressing factors that affect the health of groups of people. Even population interventions must be tailored to the context to build on existing programmes and connect people across the community.

Factors contributing to the disproportionate rural health burden are often public health issues, including limited access to healthcare services, health workforce shortages and underpinning social determinants such as food insecurity, low income, low levels of formal education and poor housing quality and availability. Longstanding rural health workforce shortages limit the implementation of many programmes, particularly preventive health strategies, community health assessments and effective responses to emerging local health issues. As rural communities have small populations and a smaller health workforce, there is less capacity to deal with the public health challenges they face. Many rural issues warrant public health responses, not just individual‐focused clinical management. Attention to workforce strategies, integrated care, health behaviours, healthy environments, injury, mental health, disease prevention and response to disasters all require public health efforts. Implementation requires dedicated efforts, best supported by workers trained in public health with understanding epidemiology, the social determinants of health, community engagement and commitment to health promotion.

Training health professionals to develop expertise and adopt a public health lens in their approach to disease prevention and management can assist to increase health and wellbeing outcomes. UDRHs are uniquely placed to contribute to this as they support vocational trainees, early‐career practitioners and assist skill development of the locally based health workforce through hosting undergraduate and postgraduate students. Public health training can empower local rural health practitioners to design and implement context‐specific interventions and to lead initiatives reflecting the values, needs and strengths of their communities. This is particularly important as rural clinicians often struggle to manage high clinical workloads and lack time and capacity to implement population level interventions. While addressing underpinning social, economic, environmental, commercial and cultural determinants of health is incredibly challenging from within the clinical environment of rural and remote clinics, working with trained public health practitioners could reduce the demands on rural clinicians in improving the health of patients they see.

Rural chronic condition disparities are often underpinned by issues of cost and poorer access to tailored information and a healthy environment. The burden of chronic diseases requires attention to addressing the underlying determinants through dedicated public health efforts to support behaviour change and contribute to socially inclusive, cohesive communities [9]. Clinical care is grounded in individualism yet changes in social and environmental risks operating across whole populations produce more improvement in population health than focusing interventions on high‐risk individuals [10]. With increasing use of telehealth, clinicians are less present in the community so their contribution of intellectual and capacity expertise is eroded as they no longer contribute to the local social environment, limiting their advocacy within and from the community for the essential resourcing and infrastructure required for improved health. As rural health workforces dwindle, local communities have even less voice and power to demand attention for a fair go.

A genuine interest in improving health equity must involve all sectors and levels of government and be not confined to the health sector. This is recognised in the Australian Government's National Preventive Health Strategy 2021–2030 [11] which explicitly acknowledges the importance of addressing health inequities and the need for prevention across the life course. Among immediate priorities flagged in the strategy are increased investment in prevention, embedding prevention in primary health care, and enhanced public health workforce training. This rarely happens in rural areas where the health sector is represented by clinic staff who are generally overworked and have little time for health‐promoting activities outside of the clinical setting. In the past, public health practitioners in rural areas have provided the ‘glue’, encouraging partnerships across organisations, working with the community and encouraging integrated approaches to address challenges and advocate for interprofessional, multidisciplinary and multisector approaches to developing innovative solutions.

## 4 Why UDRHs Are Well Positioned to Contribute Rural Public Health

UDRHs have persistently promoted holistic health by embedding themselves in local communities to address physical, mental, emotional, social and cultural well‐being through community partnerships, culturally safe practices and comprehensive education and training [12]. The attributes of a public health approach are exemplified and operationalised by UDRHs where staff live in and work closely with communities, build capacity, foster partnership approaches between clinical and community interventions, and consistently advocate for rural health equity and appropriate health services [13]. Their focus on data, evaluation and research uses core public health skills. UDRHs have contributed over many years to rural health issues, showing the value of what they do, working with regional health services, providing policy‐relevant evidence as part of their role in training health practitioners and applied research translation [3, 14, 15, 16].

When UDRHs were established, training students undertaking public health and health promotion degrees was embraced; by working and living together with students undertaking clinical degrees, the learning of both was enhanced [3] with tangible longer term impacts [17]. Students also undertook public health projects designed by communities that trained students how to work effectively in rural settings to improve health outcomes while simultaneously improving local health systems. However, the RHMT programme expanded funding for UDRHs in 2016 following abolition of Health Workforce Australia, which narrowed the relevant health professions to those with a clinical focus and excluded public health students. This fails to consider how rural workforce needs might be different and benefit from public health. We argue that the broader health workforce ecosystem would benefit from specific inclusion of public health into RHMT training through upskilling rural clinicians, developing their competencies in population health and enabling public‐health career pathways. UDRHs should have a clear role in supporting better health and care delivery by working across the health workforce pipeline and building greater understanding and responsibility in delivering equity focussed health professionals. We call for public health to be included as part of interprofessional learning under RHMT programme. UDRH programmes and research could be enhanced to expedite achieving national targets for prevention in rural areas; this fits well with the UDRH's focus on community, relationships, health equity and using data to improve all aspects of rural health both within and outside of clinical settings [3, 13, 17, 18].

## 5 Special Considerations for First Nations Communities

The challenges First Nations Australians experience with respect to their health are profound and have been repeatedly described over many years [19]. Their life challenges are not confined to rural areas, yet their health problems are particularly stark in more remote areas where First Nations people are disproportionately represented. In these settings, the impact of the social determinants of health, including ongoing exclusionary practices and outright racism, is glaring [20] and public health efforts along the lines of the Ottawa Charter are required [21]. Although the catalyst to Closing the Gap was the substantial differentials in life expectancy and health for First Nation Australians, the need for a broader approach to changing this is recognised in the key indicators of the Close the Gap targets, such as education, employment, housing, economic participation and justice [22]. To holistically address the underlying problems requires a public health approach with coordination of actions driven by communities, the inclusion of culturally appropriate processes, long‐term vision and going beyond expertise in a single area, as identified The National Aboriginal and Torres Strait Islander Health Plan 2021–2031 [23].

Public health training that occurs in rural and remote First Nations settings will emphasise approaches that are culturally safe, community‐led and grounded in First Nations knowledge systems. These understandings will assist efforts to close the gap, particularly in amplifying local voices in rural communities [24]. The National Preventive Health Strategy identifies First Nations and Rural and Remote communities as priority populations with a stated measure of success: ‘The public health workforce is ‘future proofed’ through the enhancement of the availability, distribution, capacity and skills of the workforce’. (11)p.46. Significant support to develop First Nations public health focussed practitioners from, in, and for rural Australia is long overdue. UDRHs have demonstrated longstanding commitment to improving First Nations health, particularly using public health approaches that increase access to health care, blend First Nations and Western knowledges, address racism and other determinants, and increase capacity in First Nations health [25, 26].

## 6 Policy and System‐Level Support

The causes of poor health are understood at the national policy level with multiple strategies, such as tobacco, alcohol, obesity and mental health relying on public health initiatives to effect population level change. It is *not* that more policies are needed to support rural; rural often gets mentioned in national strategies; however, the investment into resourcing for implementation at local levels in rural regions is inadequate. What we need is commitment to resourcing and growing rural public health with required implementation of proposed interventions through a locally based public health workforce that engages with community, health services and clinicians. A missing piece in implementation is a rural training pipeline that recognises public health students as an essential component of the contemporary, multidisciplinary healthcare team.

Therefore, we call on the Australian Government to recognise the essential value of public health within the RHMT programme as part of a comprehensive approach to achieving equity in rural health workforce. Encouraging UDRH support of public health focused students alongside clinical students would acknowledge the critical role of public health. We know from experience that public health students in rural settings utilise their skills to implement health prevention strategies and can help coordinate available local rural care, enhance systems between rural hospitals and primary care, develop locally tailored approaches to prevention, and tackle public health issues for First Nations and other specific groups. In this way, commitment through public health students provides the enablement which can supplement the capacity of rurally based healthcare professionals to address some of the ongoing major health issues occurring at population level. UDRHs are keen to provide evidence of the benefits that these students deliver in underserved rural areas where public health knowledge and capacity are often low. Achieving health equity requires significant investment in rural public health to address the social drivers of health; allowing public health as a discipline for support within UDRHs is a low‐cost strategy towards equity and health gain. Attention to a relevant rural public health workforce also must be included in national and state workforce strategies and public health training integrated into rural health service planning. Efforts to measure dedicated resources invested in rural and remote public health and to capture their activities are needed and could provide a salutary measure now and over time.

## 7 Conclusion

Australia's rural and remote communities deserve equitable access to health and wellbeing. Building resilient rural health systems requires sustained investment in people, organisations, and networks, not just infrastructure. Dedicated public health practitioners implement population‐level initiatives in local contexts. Training health students to develop clinical skills alongside students who are public health focussed enriches learning and the capacity to achieve more robust solutions to local problems. RHMT should not stand in isolation from national strategies as a key enabler for these strategies in rural and remote Australia.

The Ottawa Charter highlighted the need to reorient health services, shifting healthcare focus from purely curative/clinical care to prevention and health promotion, expanding the sector's role to include supporting community well‐being by addressing social determinants, fostering intersectoral links (social, political, economic factors), investing in primary care, and changing professional training towards empowering individuals for healthier lives, moving beyond ‘downstream’ treatment to ‘upstream’ health improvement. These approaches are essential for greater health equity in rural communities [21]. By adopting flexible, community‐driven training models and supporting local leadership, Australia can build a rural health workforce that is capable, committed, connected to the communities it serves, and oriented to the broader issues that underpin population health. A greater focus on public health in rural areas, including enabling public health students to be supported in rural areas as part of their training, can be readily implemented immediately, is effectively cost neutral to government, and could begin a serious look at greater investment into public health in rural Australia.

## Author Contributions

**Lisa Bourke:** conceptualization, investigation, methodology, writing – review and editing, project administration, resources, writing – original draft. **Sandra C. Thompson:** conceptualization, project administration, writing – review and editing, writing – original draft, resources, methodology, investigation.

## Funding

No specific funding was provided for this commentary. Both authors are employed by universities in rural academic centres funded under the Australian Government's Rural Health Multidisciplinary Training Programme.

## Disclosure

The authors are directors at two Australian University Departments of Rural Health. While employed by universities, the University Department of Rural Health programmes where the authors are employed are funded through the Australian Government Department of Health, Disability and Ageing under the Rural Health Multidisciplinary Training Programme.

## Conflicts of Interest

The authors declare no conflicts of interest.

## Supporting information


**Data S1:** ajr70191‐sup‐0001‐supinfo.docx. **Supporting Informartion**.

## Data Availability

Data sharing not applicable to this article as no datasets were generated or analysed during the current study.
