# Supplementary material for: A Role for Public Health Training Through the Funding of University Departments of Rural Health
Source: Aust J Rural Health. 2026 Apr 9;34(2):e70191. doi: 10.1111/ajr.70191 (PMC13066722; doi:10.1111/ajr.70191)
Supplement: Supplementary file 1 — Data S1: ajr70191‐sup‐0001‐supinfo.docx. Supporting Informartion. [file AJR-34-0-s001.docx]

**Supplementary Paragraph**

**Funding Statement**

No specific funding was provided for this commentary. Both authors are employed by universities in rural academic centres funded under the Australian Government’s Rural Health Multidisciplinary Training Program.

**Supplementary Paragraph**

Professors Sandra Thompson and Lisa Bourke are Directors of University Departments of Rural Health in the Midwest/Pilbara regions of Western Australian (UWA) and northern Victoria (University of Melbourne). They are committed to improved health and wellbeing of residents in rural areas and have undertaken research focusing on understanding the challenges in rural health, particularly the need to address factors underpinning poor health in rural areas health, working with First Nationals Australians and the development of the rural health workforce.

This commentary reflects their concerns at the sustained erosion of public health capacity in rural and remote Australia. This decline is evident in limited investment in health promotion and minimal attention to creating safer, healthier environments where rural residents live, work and play. Health system responses focus disproportionately on ill health and acute care with insufficient attention to the social determinants of health and building inclusive, resilient and sustainable rural communities.

While national/state health strategies routinely acknowledge the poorer health outcomes experienced by rural and remote populations, there is a lack of meaningful, long-term investment in smaller rural communities. The issue is not a lack of policy, but a persistent mismatch between need and resourcing, with limited commitment to strengthening public health and prevention in rural contexts. The authors highlight the need for dedicated investment in rural, remote and First Nations health literacy and preventive health.
